# Increasing Serotonin to Reduce Parkinsonian Tremor

**DOI:** 10.3389/fnsys.2021.682990

**Published:** 2021-07-20

**Authors:** Daniele Caligiore, Francesco Montedori, Silvia Buscaglione, Adriano Capirchio

**Affiliations:** ^1^Computational and Translational Neuroscience Laboratory, Institute of Cognitive Sciences and Technologies, National Research Council, Rome, Italy; ^2^Neurophysiology and Neuroengineering of Human-Technology Interaction Research Unit (NeXT), Campus Bio-Medico University, Rome, Italy

**Keywords:** computational neuroscience, different parkinsonian tremor types, differential equations brain modeling, patient digital twin, Parkinson's disease early diagnosis, serotonin and dopamine interplay, system neuroscience, tremor alternative treatment

## Abstract

While current dopamine-based drugs seem to be effective for most Parkinson's disease (PD) motor dysfunctions, they produce variable responsiveness for resting tremor. This lack of consistency could be explained by considering recent evidence suggesting that PD resting tremor can be divided into different partially overlapping phenotypes based on the dopamine response. These phenotypes may be associated with different pathophysiological mechanisms produced by a cortical-subcortical network involving even non-dopaminergic areas traditionally not directly related to PD. In this study, we propose a bio-constrained computational model to study the neural mechanisms underlying a possible type of PD tremor: the one mainly involving the serotoninergic system. The simulations run with the model demonstrate that a physiological serotonin increase can partially recover dopamine levels at the early stages of the disease before the manifestation of overt tremor. This result suggests that monitoring serotonin concentration changes could be critical for early diagnosis. The simulations also show the effectiveness of a new pharmacological treatment for tremor that acts on serotonin to recover dopamine levels. This latter result has been validated by reproducing existing data collected with human patients.

## 1. Introduction

Resting tremor is one of the most disabling features of Parkinson's disease (PD). People affected by resting tremor exhibit uncontrollable movements involving a body part, for example, the arm, when at rest. Tremor tends to decrease or stop when they deliberately move the affected part of the body (Deuschl et al., 2000; Kalia and Lang, 2015). Like other PD motor features, tremor is thought to result primarily from the death of dopamine-producing cells in the substantia nigra pars compacta (SNc), an area in the midbrain mainly targeting the striatum. This area is the main input gate of the basal ganglia, subcortical nuclei critical to managing motor behavior. Thus, a consistent reduction of striatal dopamine levels causes malfunctioning of the basal ganglia circuits that, in turn, may contribute to the emergence of tremor at rest (Pare et al., 1990; Wichmann and DeLong, 1999; Dovzhenok and Rubchinsky, 2012).

Based on this evidence, drug therapies for tremor often aim at recovering dopamine levels (Deuschl et al., 2000; Caligiore et al., 2019). However, while these approaches seem to produce amelioration for most PD motor dysfunctions, they generate variable responsiveness for resting tremor (Helmich et al., 2012; Wu and Hallett, 2013; Connolly and Lang, 2014). The lack of dopamine-based therapies consistency with tremor could be explained by considering that based on the dopamine response, PD resting tremor can be divided into various partially overlapping phenotypes that may be associated with different pathophysiological mechanisms (Zach et al., 2020). These mechanisms might be produced by a cortical-subcortical network involving areas traditionally not directly related to PD. Several data support this framework (Obeso et al., 2010; Caligiore et al., 2016, 2017b; Helmich, 2018).

The membrane properties of the basal ganglia cells support pacemaking (Surmeier et al., 2005) but do not produce tremor oscillations in healthy basal ganglia circuits. In contrast in PD, tremor-related activity (i.e., neural activity in the tremor frequency band, correlated with the tremor movement) was observed in the globus pallidus (Hutchison et al., 1997) and subthalamic nucleus (STN) (Levy et al., 2000), in the thalamus (Thal) (Lenz et al., 1994), and in the cortex (Timmermann et al., 2003). Lesions in different parts of the basal ganglia-thalamo-cortical network [in the cortex (Deuschl et al., 2000), globus pallidus, and Thal (Mitchell and Ostrem, 2020), in the STN (Alvarez et al., 2005)] suppress tremor. The fact that breaking the loop at multiple sites leads to the same effect, tremor suppression, suggests that *the loop itself*, more than any of its parts, contributes to tremor generation.

The tremor network can also involve the dorsal raphe nucleus (DRN), which could modulate the release and concentration of dopamine through the serotonergic projections it sends to the striatum (Bara-Jimenez et al., 2005; Di Matteo et al., 2008; Politis and Niccolini, 2015). It has been recently shown that aside from the dopaminergic dysfunction, in PD, there is a progressive loss of serotonergic terminals that has a slower pace and *begins earlier* than the dopaminergic one. This serotonergic dysfunction has been associated with the development of both non-motor and motor symptoms (De La Fuente-Fernndez et al., 2001; Lindgren et al., 2010; Politis and Niccolini, 2015) including tremor (Jankovic, 2018; Pasquini et al., 2018).

Overall, the vast range of conditions provoking or relieving tremor supports the involvement of a complex brain network, including both cortical and subcortical areas. Various dysfunctions within this network can produce *different types of tremors*. One mainly due to a *direct* dopaminergic system deficiency and for which dopamine-based actions work. Others involving the dopaminergic system and other brain areas, for which dopamine-based treatments are less effective (Pasquini et al., 2018; Zach et al., 2020).

Starting from this system-level perspective, we propose a bio-constrained computational model to study the neural mechanisms underlying a possible type of PD tremor: the one mainly involving the serotonergic system. The model reproduces the interactions between basal ganglia direct (DP) and indirect pathways (IPs), primary motor cortex (M1) and Thal (Marreiros et al., 2013; Hintzen et al., 2018), and the critical role of the DRN, the brain region producing serotonin, on the basal ganglia circuits (Padovan-Neto et al., 2020). More in detail, the model reproduces the excitatory and inhibitory influences between these brain areas through an ordinary differential equations system (Reed et al., 2013). Moreover, it drives the movement of a simulated 2D anthropomorphic robotic arm, reproducing the main features of the human arm (Katayama and Kawato, 1993; Caligiore et al., 2014).

The simulations run with the model demonstrate that serotonin could affect dopamine levels to maintain homeostasis between the DP and IP signals (Di Giovanni et al., 2002; Guiard et al., 2008; Reed et al., 2013; Sgambato-Faure and Tremblay, 2018). When there is a dopaminergic dysfunction, the model shows that the serotonin-based *compensation mechanism* is not able by itself to bring the system back to physiological dopamine levels. This result agrees with recent studies (Roussakis et al., 2016; Jiménez-Sánchez et al., 2019). However, the model suggests that the compensation mechanism can nevertheless mitigate the progression of the disease in the very early stages, before the manifestation of overt tremor. The model also shows that serotonergic dysfunctions affect striatal dopamine release, indirectly contributing to the emergence of a serotonin-related tremor phenotype. Overall, these results could be critical to devising new early diagnosis strategies based on serotonin monitoring (Wilson et al., 2019).

The model allowed us to study the effect of a possible new drug therapy that builds on the serotonin-compensatory role to recover dopamine levels in simulated patients. This drug produces a boosting of the serotonin physiological compensatory effect that contributes to reduce tremor. This latter result has been validated by reproducing existing data collected with human patients, showing that a serotonin increase could produce a tremor decrease (Qamhawi et al., 2015).

## 2. Materials and Methods

### 2.1. Model Architecture

**Figure 1:**
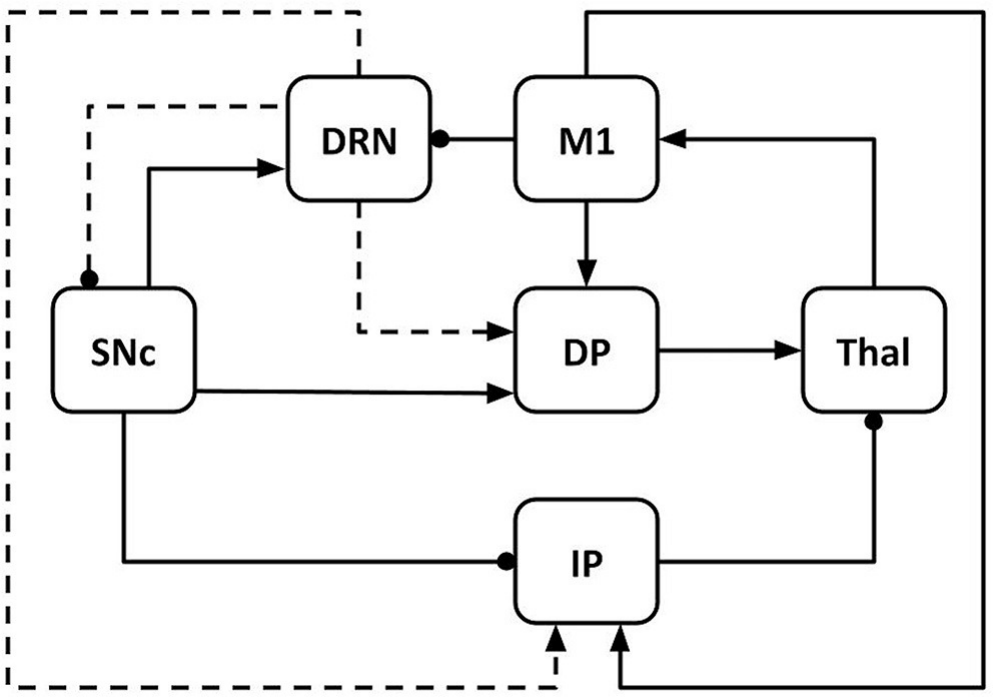
Model architecture including primary motor cortex (M1), thalamus (Thal), dorsal raphe nucleus (DRN), substantia nigra pars compacta (SNc); basal ganglia direct pathway (DP); basal ganglia indirect pathway (IP). The connections linking different components can be excitatory (arrows) or inhibitory (lines ending with dot). The dashed lines indicate the effects of serotonergic projections from DRN to SNc, DP, and IP. Through these connections, serotonin modulates the dopamine release in the system.

The model architecture reproduces the dynamical interaction between six components (Figure 1): M1, Thal, DRN, SNc, DP, and IPs. DP reproduces the overall activity of the striatum and the internal portion of the globus pallidus, whereas IP reproduces the activity of the circuit involving the external globus pallidus (GPe) and the STN (Smith et al., 1998; Gurney et al., 2001; Haber, 2003).

The model components communicate through excitatory and inhibitory connections. M1 receives excitatory input from the Thal (Guillery and Sherman, 2002; Bosch-Bouju et al., 2013; Planetta et al., 2013; Stephenson-Jones et al., 2016) and sends excitatory output to DP (striatum) and IP (STN) (Bolam et al., 2000; Caligiore et al., 2019; Paraskevopoulou et al., 2019). M1 also produces an inhibitory effect on DRN (through other cortical areas not reproduced here for simplicity, e.g., prefrontal cortex) (De Simoni et al., 1987; Monti, 2011; Lopes et al., 2019; Sargin et al., 2019). DRN receives excitatory signals from SNc and sends serotonergic projections to SNc (inhibitory), DP, and IP (both excitatory). Through these connections, it modulates the dopamine (DA) release to DP and IP (Vertes, 1991; Di Matteo et al., 2008). Thal receives input from the basal ganglia DP (excitatory connections) and IP (inhibitory connections) (Alexander et al., 1986; Smith et al., 1998; Middleton and Strick, 2000; Gerfen and Surmeier, 2011; Caligiore et al., 2013). SNc produces a DA release that has an excitatory effect on DP and an inhibitory effect on IP (Cachope and Cheer, 2014).

### 2.2. Model Equations

The following differential equations system simulates the activity of the model components and their dynamical interactions:

(1) $$\begin{array}{l} \overset{\cdot }{M1}=a_{1Thal}\cdot Thal-\tau_{M1}\cdot M1 \end{array}$$

(2) $$\begin{array}{l} \overset{\cdot }{Thal}=a_{2Ex}+a_{2DP}\cdot DP-a_{2IP}\cdot IP-\tau_{Thal}\cdot Thal \end{array}$$

(3) $$\begin{array}{l} \overset{\cdot }{DRN}=a_{3Ex}-a_{3M1}\cdot M1+a_{3SNc}\cdot SNc-\tau_{DRN}\cdot DRN \end{array}$$

(4) $$\begin{array}{l} \overset{\cdot }{5-HT}=a_{4DRN}\cdot DRN-\tau_{5-HT}\cdot 5-HT \end{array}$$

(5) $$\begin{array}{l} \overset{\cdot }{SNc}=a_{5Ex}-a_{5DRN}\cdot DRN-\tau_{SNc}\cdot SNc \end{array}$$

(6) $$\begin{array}{l} \;\overset{\cdot }{DA}=G\cdot 5-HT\cdot SNc-\tau_{DA}\cdot DA \end{array}$$

(7) $$\begin{array}{l} \overset{\cdot }{DP}=a_{7Ex}+a_{7DA}\cdot DA-\tau_{DP}\cdot DP \end{array}$$

(8) $$\begin{array}{l} \overset{\cdot }{IP}=a_{8Ex}-\tau_{IP}\cdot IP+\frac{\alpha_{IP}\cdot \sin\left(f_{IP}\cdot t\right)}{DA} \end{array}$$

The equations reproduce: the influence of Thal on M1 (Equation 1); the effects of the DP and IP on Thal activity (Equation 2); the modulation of DRN by M1 and SNc (Equation 3); the serotonin (5-HT) release from the DRN (Equation 4); the influence of DRN on SNc activity (Equation 5); the DA release from SNc in response to 5-HT concentration (Equation 6); the influence of DA on medium spiny neuron (msn) in the DP (Equation 7); and the influence of the GPe-STN loop and DA on IP (Equation 8).

We obtained this system by critically manipulating the equations proposed by Reed et al. (2013). In particular, we introduced two innovations critical to investigate the role of serotonin in PD tremor: (i) a more detailed IP equation (Equation 8) to simulate the oscillatory behavior affecting the STN-GPe loop (Nambu et al., 2002; Stanford, 2003; Surmeier et al., 2005); (ii) a bio-constrained 2D dynamic arm, allowing to record the effects of serotonin increase on overt tremor (Qamhawi et al., 2015). Although the reciprocal connections linking GPe and STN can produce and maintain low-frequency rhythms (≤5*Hz*) (Plenz and Kital, 1999; Terman et al., 2002), they do not produce tremor oscillations in healthy basal ganglia circuits. In PD, the DA loss induces an excessive synchronization of the GPe-STN oscillatory activity, contributing to generate and propagate abnormal tremor-related oscillations over the striatal-thalamo-cortical system (Nini et al., 1995; Guillery et al., 1998; Boraud et al., 2005; Gatev et al., 2006; Özkurt et al., 2011).

These equations allow studying the modulation between the various components of the model with simple linear functions, except for the oscillatory term (Equation 8), and for the term that represents the DA release in the striatum (Equation 6). Moreover, DA has an excitatory effect on the DP activity (Equation 7) and an inhibitory effect on IP (Equation 8). These two different types of dopaminergic modulations reflect what happens in the real basal ganglia (Cachope and Cheer, 2014; Fiore et al., 2015). Finally, each equation has a decay term, ensuring that the variable (firing rates or concentrations) goes to zero in the absence of input.

### 2.3. Dynamic Arm

**Figure 2:**
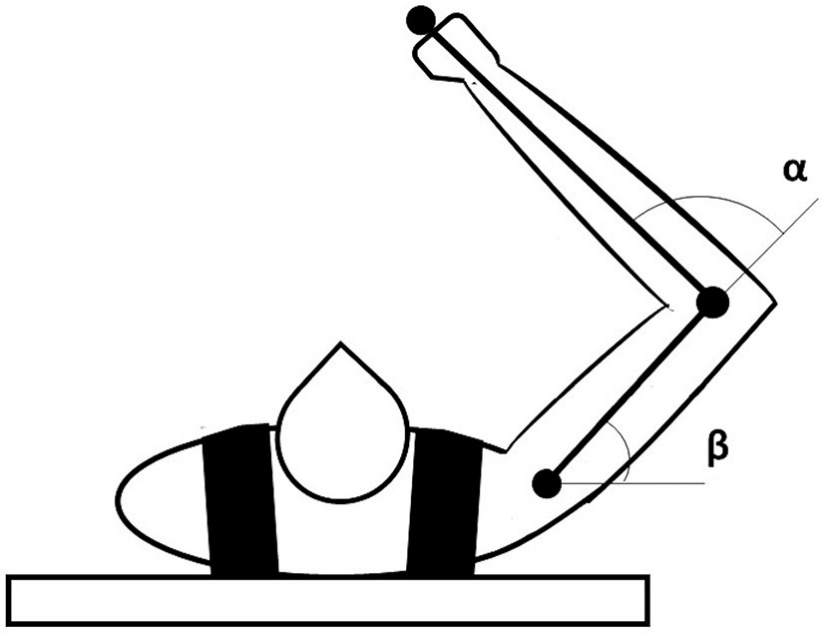
Representation of the dynamic arm used to test the model motor behavior. The task space is a horizontal plane in which the arm is free to move according to the inputs received from M1.

The model controls a simulated 2D dynamic arm (Flash and Hogan, 1985) implemented with the *realistic biomechanical parameters* proposed in Katayama and Kawato (1993) (as shown in this study for more details on the arm equations) (Figure 2). We numerically integrated the dynamical arm equations using a Runge-Kutta fourth-order method with an integration step set to 0.01. The arm shoulder is anchored at [0.0, 0.0] coordinates of the workspace. The range of variation of the shoulder (β) and elbow (α) angles are [−60.0°, 150.0°] and [0.0°, 180.0°], respectively. The arm movements are driven by M1 signals obtained in three different model conditions: before DA dysfunction (pre-lesion), after DA dysfunction (post-lesion), and after serotonergic treatment (post-treatment). Minimum and maximum values of M1 activity are suitably scaled to get, respectively, minimum and maximum values of the elbow and shoulder angles.

We used a proportional derivative controller (PD_controller_) to capture some muscular visco-elastic properties of the human arm (Caligiore et al., 2014). This mathematical model computes the torques vector (*T*) necessary to move the arm joints, starting from the desired angles supplied by M1 (*A*_*des*_), the current angular position (*A*_*cur*_), and the current angular velocity ($\overset{\cdot }{A_{cur}}$):

(9) $$\begin{array}{l} T=K_{p}\left(A_{des}-A_{cur}\right)-K_{d}\overset{\cdot }{A_{cur}} \end{array}$$

where *K*_*p*_ is the vector containing the values of the PD proportional constant for the shoulder and elbow joints (shoulder = 20.0 Nm/rad; elbow = 10 Nm/rad), whereas *K*_*d*_ is the vector containing the values of the PD_controller_ damping constant (shoulder = 1.5 Nm/rad; elbow = 1.0 Nm/rad). These values were chosen taking into account human arm visco-elastic properties (An et al., 1981).

### 2.4. Simulation Settings

The model was developed using the Python programming language. The code is available here https://github.com/ctnlab/serotonin_PD_tremor_model.

#### 2.4.1. Tuning the Model Parameters

Table 1 summarizes the values of the model parameters and their biological meaning. The values of the model parameters were set to obtain a steady-state for the variables of the equation similar to those found in experimental observations (Feldman et al., 1997; Segovia et al., 1997; Jones et al., 1998; Knobelman and Lucki, 2001; Mahon et al., 2006; Ohara et al., 2007; Shimamoto et al., 2013) (see Table 2). In particular, the parameters settings were done through an automatic optimization procedure, namely a “genetic algorithm” (Fortin et al., 2012; Caligiore et al., 2017a). The algorithm went through optimization cycles called “generations”. The parameters search started from the values used in Reed et al. (2013). For each generation, a “population” of candidate solutions corresponding to different possible sets of model parameters was “evolved” to (1) minimize the mean absolute percentage error (*APE*) between the model steady-state values and those drawn from the experimental observations. A low value of such error indicated that the activity exhibited by the model was similar to the data collected from the real subjects; (2) minimize the oscillation in the healthy model (*meanPhys*); (3) maximize the oscillation in the damaged model (*meanPark*). Each “generation” explores 30 candidate parameter populations, and hence models (during 300 generations of the genetic algorithm), to get a *meanAPE* = 0.003 (*fitness* = 0.9969), a value that guaranteed a good matching between the simulated and the real data; *meanPhys* = 0.0053 (*fitness* = 0.9947) to guarantee a low value of physiological oscillation; and (*meanPark* = 0.0051) to predict oscillation amplitude observed in PD subjects (*fitness* = 0.9949). The parameters shown in Table 1 are the best ones obtained with this procedure.

**Table 1:** Parameters of the equations.

**Parameters**	**Value**	**Explanation**
*a*_1*Thal*_	1.3655 Hz	Influence of Thal to M1
τ_*M*1_	0.9330 s	Decay constant of cortical neurons
*a*_2*Ex*_	1.5985 Hz	External drive to Thal
*a*_2*DP*_	3.7502 Hz	Excitation of DP to Thal
*a*_2*IP*_	2.1032 Hz	Inhibition of IP to Thal
τ_*Thal*_	0.2547 s	Decay constant of Thal neurons
*a*_3*Ex*_	6.6911 Hz	External drive to DRN
*a*_3*M*1_	0.1671 Hz	Inhibition of DRN by M1
*a*_3*SNc*_	0.010 Hz	Excitatory influence of SNc to DRN
τ_*DRN*_	1.6829 s	Decay constant of DRN neurons
*a*_4*DRN*_	1.1951 Hz	Influence of DRN to 5-HT release in the striatum
τ_5−*HT*_	1.9949 s	Decay constant of 5-HT in the striatum
*a*_5*Ex*_	60.4428 Hz	External drive to SNc
*a*_5*DRN*_	13.6055 Hz	Inhibition of SNc by DRN
τ_*SNc*_	9.2357 s	Decay constant of SNc neurons
*G*	0.8083	Influence per nM of 5-HT on DA release in the striatum
τ_*DA*_	1.1173 s	Decay constant of DA in the striatum
*a*_7*Ex*_	2.4290	cortical input to msn in DP
*a*_7*DA*_	0.1544 Hz	Influence of DA in msn in DP
τ_*DP*_	1.0648 s	Decay constant of msn in DP
*a*_8*Ex*_	1.1229	cortical input to msn in IP
*f*_*IP*_	6.1493 Hz	Oscillation frequency
*alpha*_*IP*_	7.3801	Effectiveness of DA in IP
τ_*IP*_	1.0283 s	Decay constant of msn in IP

**Table 2:** Variables of the equations.

**Variable**	**Steady state**	**Explanation**	**Source**
*M*1	23.6 Hz	Cortical neurons (FR)	Shimamoto et al., 2013
*IP*	1.88 Hz	Striatal spiny neurons in IP (FR)	Mahon et al., 2006
*DP*	1.85 Hz	Striatal spiny neurons in DP (FR)	Mahon et al., 2006
*Thal*	17.5 Hz	Thalamic neurons (FR)	Ohara et al., 2007
*DRN*	1.41 Hz	Dorsal raphe nucleus neurons (FR)	Feldman et al., 1997
*SNc*	4.47 Hz	Substantia nigra pars compacta neurons (FR)	Feldman et al., 1997
*5-HT*	0.846 nM	Serotonin in the striatum (C)	Knobelman and Lucki, 2001
*DA*	2.72 nM	Dopamine in the striatum (C)	Segovia et al., 1997; Jones et al., 1998

*FR, firing rate; C, concentration*.

#### 2.4.2. Simulation Trials

The model equations are numerically integrated with an integration step Δ*t* = 0.1 (i.e., one simulation step corresponds to 0.1 *s* of real-time). The simulation runs for 300 *s* (3,000 simulation steps). At the beginning of the simulation, the shoulder and elbow angles of the arm are set to the starting position (α = 0°; β = 0°). Within the simulation time, we monitor the changes in the model components (i.e., activity of brain areas and neuromodulators concentrations) in five-time windows that we called “simulation trials”: the first one lasts 75 *s* and it is used to simulate the behavior of the health system; the second one (75, 125) *s* is used to study the effects of the DA lesion (occurring at 75 *s*); the other three remaining trials (the first two lasting 50 *s* each, the last 75 *s*) are used to investigate the effects of increasing doses of the simulated serotonin-based treatment.

#### 2.4.3. Experiment Repetitions

We collected data from 20 different simulated subjects, obtained by running the model with distinct seeds of a pseudo-random number generator, which in turn causes different values of a noise signal acting on the model. In particular, we added to the initial values of the variables of the equations (Table 2), a small random number drawn from a normal distribution having mean zero and SD set to the initial value times, a number increasingly chosen in the range (0.01, 0.02) (0.01 for the subject 1, 0.02 for the subject 20, with an increasing step set to 0.0005). At the same time, we added to the values of the parameters of the equations (Table 1), a small random number drawn from a normal distribution having mean zero and SD set to the value of the parameter times, a number increasingly chosen in the range (0.01, 0.02) (increasing step still set to 0.0005). In this way, we exploited the capacity given by the simulation approach to producing many subjects with low cost to have higher statistical reliability of the results.

## 3. Results

This section presents the results obtained with the model and directed to (i) support the 5-HT role in maintaining homeostasis between DP and IP by affecting DA release (Reed et al., 2013; Sgambato-Faure and Tremblay, 2018); (ii) demonstrate that 5-HT dysfunctions can indirectly contribute to the emergence of tremor by producing a DA decrease; (iii) study the effect of a new pharmacological treatment for tremor that acts on 5-HT to recover DA levels. This latter result was validated by reproducing data collected with human patients showing that 5-HT enhancement could reduce tremor (Qamhawi et al., 2015).

### 3.1. Serotonin Compensates Dopamine Loss

To investigate how 5-HT affects DA release, we introduced an SNc malfunctioning. At the beginning of the simulation, the model has no dysfunctions. After 75 *s*, we introduced an SNc impairment by increasing the value of τ_*SNc*_ (+25%) (Equation 5). This action produces an SNc activity decrease approximating the effects of a neural loss. At the same time, we increase the value of α_*IP*_ (+11%) (Equation 8), simulating an efficacy decrease of the DA receptors that regulate the oscillatory component of IP.

**Figure 3:**
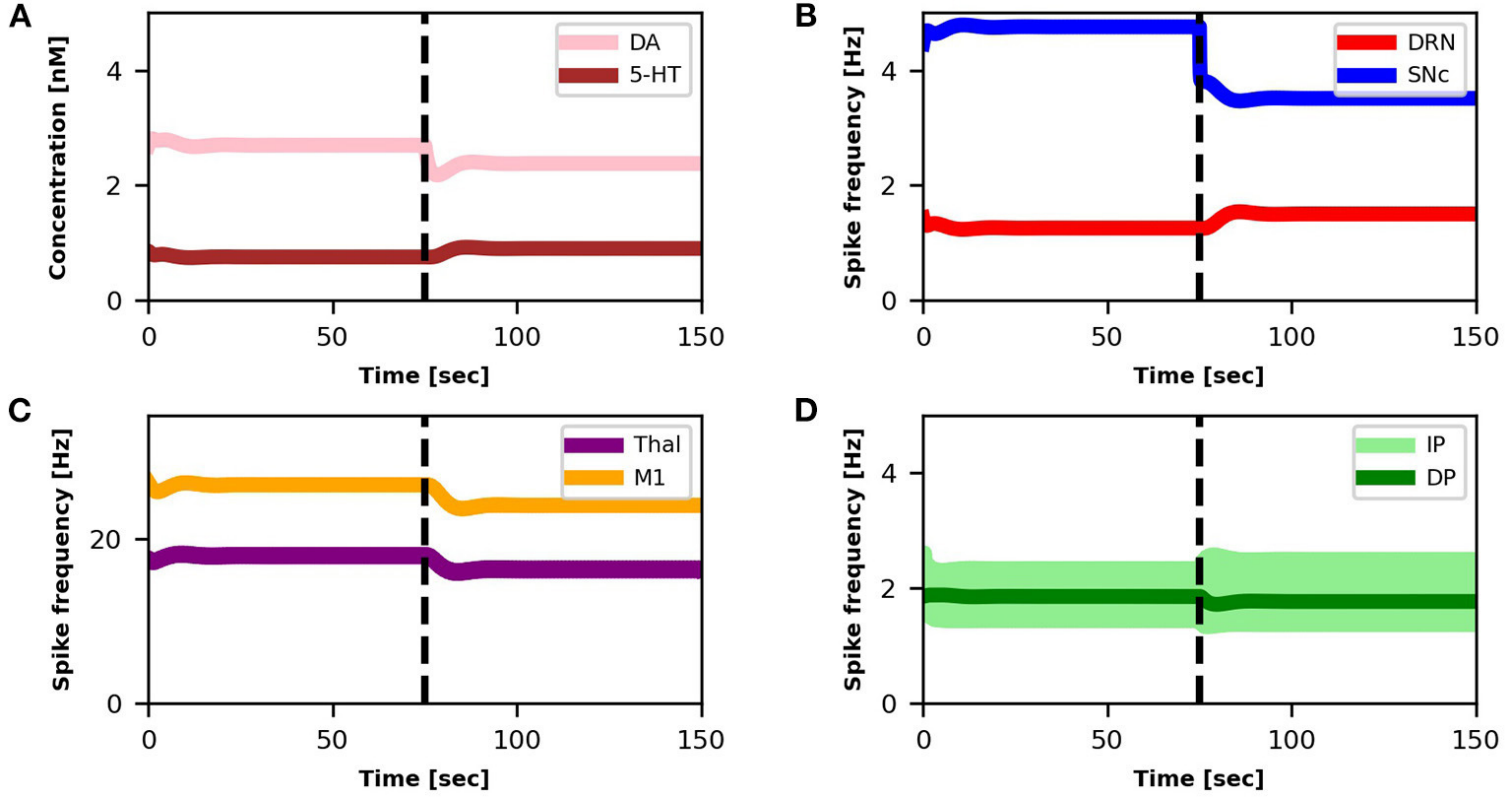
Relationship between activities of model components and changes in DA and 5-HT concentrations. The dotted vertical line represents the timing of the lesion in the dopaminergic circuits (τ_*SNc*_ + 25%, α_*IP*_ + 11%). The four graphs represent: **(A)** the concentration levels of 5-HT (brown line) and DA (pink line); **(B)** the spike frequency of DRN (light red line) and SNc (light blue line); **(C)** the spike frequency of Thal (orange line) and M1 (purple line); **(D)** the spike frequency of IP (light green line) and DP (dark green line).

The SNc spike frequency decreases, leading to a reduction of DA concentration (Figures 3A,B). This loss produces a chain of effects: Thal activity decreases (Figure 3C) because DP activity goes down whereas IP activity goes up (Figure 3D); M1 activity decreases (Figure 3C) because it directly depends on Thal activity. Lower activation of M1 produces a lower inhibition of DRN that, as a consequence, increases its activity (Figure 3B). Finally, higher activity of DRN supports an increase of 5-HT release that, in turn, *partially recovers* the DA level (Figure 3A).

### 3.2. Serotonin Dysfunctions Produce Dopamine Loss

**Figure 4:**
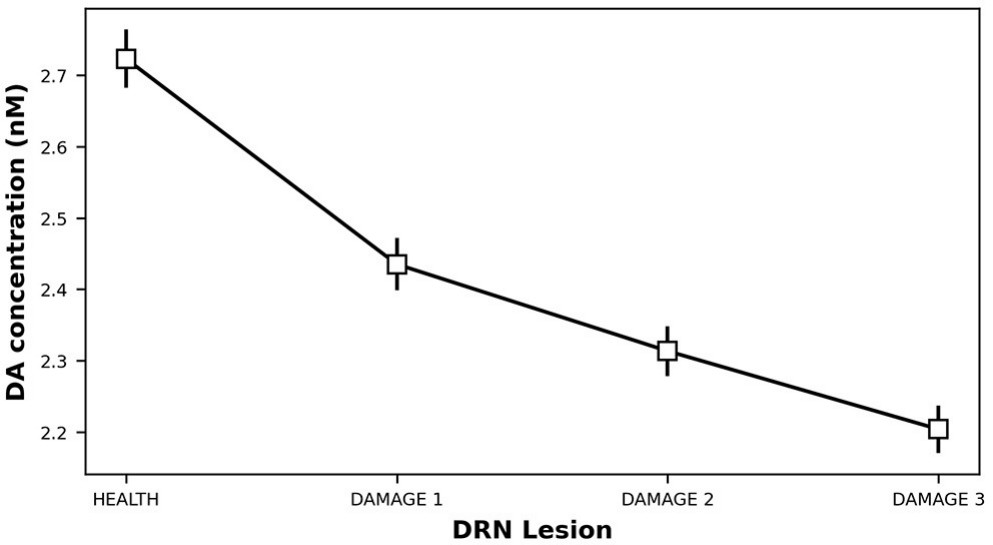
Dopamine concentration vs. 5-HT release impairments. DAMAGE1 = τ_*DRN*_ + 40%; DAMAGE2 = τ_*DRN*_ + 60%; DAMAGE3 = τ_*DRN*_ + 80%.

**Figure 5:**
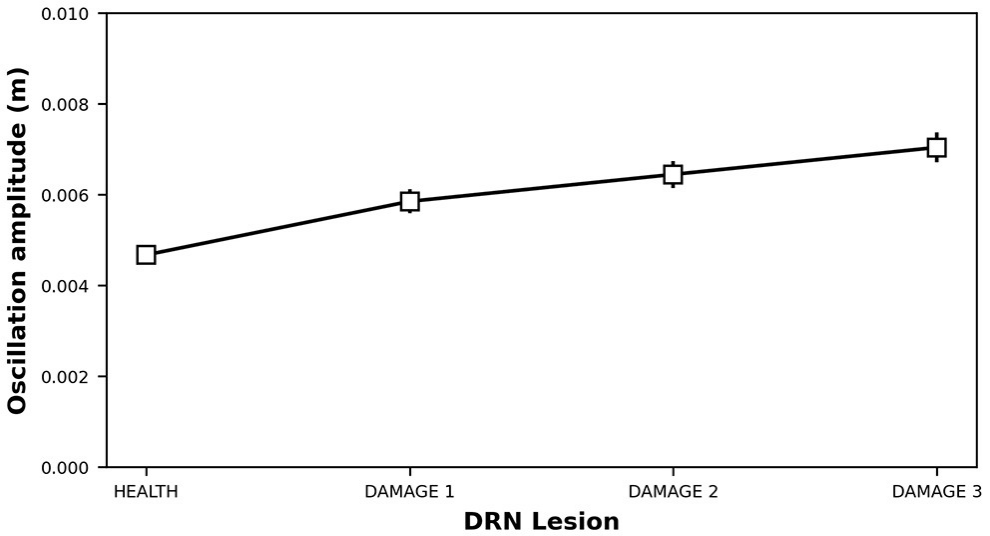
Oscillation induced by 5-HT release impairments. DAMAGE1 = τ_*DRN*_ + 40%; DAMAGE2 = τ_*DRN*_ + 60%; DAMAGE3 = τ_*DRN*_ + 80%.

Starting from a healthy system, we produced a 5-HT impairment by gradually increasing the τ_*DRN*_ parameter, approximating the effects of a DRN neural loss (Equation 3). Figure 4 shows that as we increase the degree of the simulated 5-HT impairment, the DA concentration in the striatum drops down. As a result, the oscillatory component of IP increase (Equation 8), feeding the emergence of an overt tremor (Figure 5). This outcome supports the hypothesis that 5-HT dysfunctions might indirectly contribute to the emergence of tremor.

The results shown in Figures 4, 5 were statistically validated through a repeated-measures ANOVA with the condition (HEALTH, DAMAGE 1, DAMAGE 2, and DAMAGE 3) as a within-subjects factor. The Bonferroni *post hoc* test was also conducted on significant interactions, and the Greenhouse-Geisser correction was used in the case of violations of sphericity. The ANOVA revealed that there is a statistically significant difference between DA concentrations in different conditions [*F*_(1,016−19,299)_ = 61,683,499, *Cohen's f* = 0, *p* < 0.001, power = 1]. Moreover, the *post hoc* analysis showed that the health condition (i.e., with no DRN damage) is significantly different from the other three conditions, with a greater DA concentration (level of DA concentration: 2,723 vs. 2,435 vs. 2,313 vs. 2,204 nM, respectively). Furthermore, the DA concentration decreases with the damage entity, the greater the DRN damage, the lower the DA concentration. Similarly, the ANOVA showed that there is a statistically significant difference between the tremor rate in different conditions [*F*_(1,011−19,204)_ = 6,967,198, *Cohen's fr* = 0, *p* < 0.001, power = 1]. The *post hoc* analysis confirmed that the health condition is significantly different from the other three conditions, the tremor, in this case, is reduced with respect to the DRN damage conditions. Furthermore, the greater the DRN damage, the greater the tremor.

### 3.3. A New Treatment for Tremor Based on Serotonin Increase

**Figure 6:**
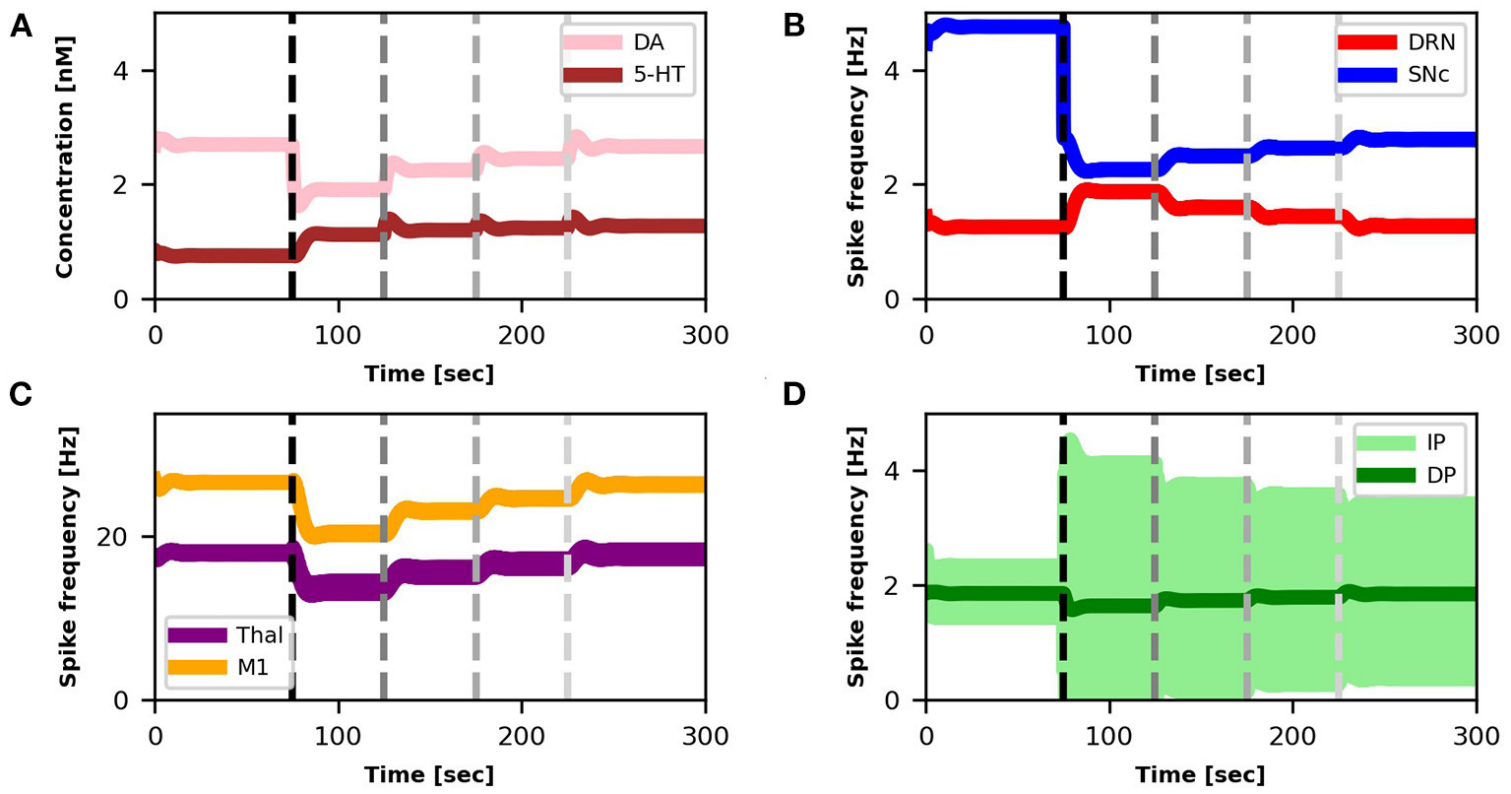
Relationship between activities of model components and changes in DA and 5-HT concentrations after SNc damage and selective serotonin inhibitors (SSRIs) simulated treatment. The first dotted vertical line (black) represents the timing of the lesion in the dopaminergic circuits (τ_*SNc*_ + 70%, α_*IP*_ + 333%). The other three dotted vertical lines (gray scale) indicate the beginning of different serotonin-based drug (SSRIs) treatment periods with different doses of the treatment (τ_5−*HT*_ − 20%, τ_5−*HT*_ − 30%, τ_5−*HT*_ − 40%). Color code and graphs displacement as Figure 3.

**Figure 7:**
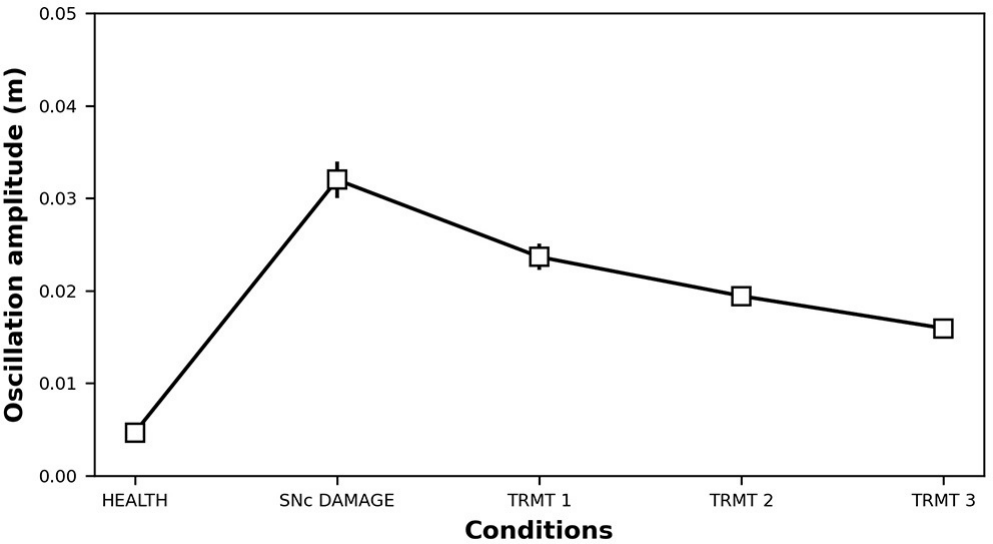
Oscillation amplitude of the bio-mimetic arm before and after the DA impairment (τ_*SNc*_ + 70%, α_*IP*_ + 333%) and following SSRIs treatment (τ_5−*HT*_ − 20%, τ_5−*HT*_ − 30%, τ_5−*HT*_ − 40%).

In this section, we show that it is possible to build on the neural mechanisms underlying the physiological influence of 5-HT on DA release (sections 3.1, 3.2), to test a new treatment for tremor based on a 5-HT increase. Figure 6 shows the effects of this treatment at the level of neural activity, whereas Figure 7 shows the effects on tremor.

Starting from a healthy model, after 75*s* we introduced an SNc impairment by increasing the value of τ_*SNc*_ (+70%) (Equation 5) and by increasing the value of α_*IP*_ (+333%) (Equation 8). Since these impairments are stronger than those produced in section 3.1, they produce a stronger DA loss (compare Figures 6A,B vs. Figures 3A,B). In this case, the physiological compensatory mechanisms involving 5-HT in between 75 and 125*s* only allow to partially recover the DA loss, and it is not strong enough to avoid tremor (case “SNc DAMAGE” on Figure 7).

Then we boost the physiological influence of 5-HT on DA release by gradually reducing the value of τ_5−*HT*_ (Equation 4) between 125 and 300 *s*. This parameter could be a proxy of the selective serotonin inhibitors (SSRIs) effects on serotonin transporters, which carry 5-HT in the DRN pre-synaptic neurons (Serretti et al., 2006; Dogan et al., 2008; Murphy and Lesch, 2008). Thus, a gradual decrease of τ_5−*HT*_ produces an increase of 5-HT release simulating the effects of a treatment based on a gradual increase of SSRIs. More in detail, we simulated three subsequent increases of SSRIs which produce the following chain of effects: Thal activity gradually increases (Figure 6C) because DP activity gradually goes up whereas the IP activity goes down (Figure 6D); as a consequence, M1 activity increases (Figure 6C) because it directly depends on Thal activity; a greater activation of M1 produces greater inhibition of DRN that, as a consequence, decreases its activity (Figure 6B); a lower activity of DRN supports a lower inhibition of SNc (Equation 5) that, in turn, recovers the DA level reaching similar levels observed before the lesion (Figure 6A). The reduction of IP oscillatory activity together with the increase of M1 activity produce a gradual decrease of tremor (Figure 7).

Data were analyzed with repeated-measures ANOVA with the condition (HEALTH, SNc DAMAGE, TRMT 1, TRMT 2, and TRMT 3) as a within-subjects factor. The Bonferroni *post hoc* test was also conducted on significant interactions, and the Greenhouse-Geisser correction was used in the case of violations of sphericity. The ANOVA revealed that the main effect of SSRIs treatment [*F*_(1,192−22,654)_ = 3,475,671, *Cohen's f* = 0, *p* < 0.001, power = 1] is significant. The *post hoc* test showed a significant increase of tremor oscillation after the damage (0.005 vs. 0.032 m), furthermore, the treatment improves the tremor, in particular, increasing the dose decreases the tremor (0.024 vs. 0.019 vs. 0.016 m).

## 4. Discussion

In this study, we proposed a system-level computational model to investigate the role of the serotonergic system in PD tremors. The results shown in Figure 3 suggest that at the very early stage of the disease, a physiological increase in 5-HT concentration, which indirectly affects DA release, could partially recover a low reduction of DA. Even though this compensatory mechanism is not able by itself to bring DA back to physiological levels, it contributes to maintaining DA at values that do not produce overt tremor in the patient (Figure 3D). Figure 4 further supports the critical role 5-HT has in the modulation of DA concentration by showing that 5-HT impairments produce DA loss and the emergence of tremor (Figure 5). These results agree with empirical data showing that high levels of 5-HT could promote DA release from SNc dopaminergic projections (Blandina et al., 1989; Bonhomme et al., 1995; De Deurwaerdère et al., 2002). Interestingly, the tremor entity (amplitude of oscillations) produced by the 5-HT dysfunction is lower than the tremor entity obtained by the impairment of the dopaminergic system (Figure 5 vs. Figure 7). The 5-HT dysfunction, indeed, only acts on SNc DA release. By contrast, the tremor entity shown in Figure 7 is due to both the SNc DA release dysfunction (τ_*SNc*_) and the efficacy reduction of D2 DA receptors that regulate the oscillatory component of IP (α_*IP*_). For this reason, the 5-HT-based treatment only partially contributes to recover the IP health activity (Figure 6D). Hence, the model suggests that 5-HT impairments alone contribute to the emergence of weak overt tremor symptoms, like the one that one could show at the very beginning of the disease progression. To have large tremor oscillations, it is also critical to consider the IP D2 DA receptors responsiveness dysfunctions. This result agrees with data we obtained using a spiking neural network model to study the differences between tremor and patients with akinetic PD (Caligiore et al., 2019) and agrees with experiments suggesting the involvement of both 5-HT and DA dysfunctions in the progression of resting tremor (Pasquini et al., 2018).

The results summarized in Figures 6, 7 demonstrate the effectiveness of a possible treatment based on the gradual increase of SSRIs on 5-HT transporters carrying 5-HT on DRN (Serretti et al., 2006; Dogan et al., 2008; Murphy and Lesch, 2008). Figure 6 shows that the physiological increase of 5-HT concentration operating before the simulated treatment (75, 125) *s* produces a compensatory mechanism that increases the DRN activity to partially recover DA levels. By contrast, the treatment (125, 300) *s* leads to a DRN reduction, producing a higher DA level recovery and a tangible reduction of overt tremor (Figure 7).

Interestingly, the simulations show that the SSRIs treatment triggers different network dynamics than those involved in the 5-HT physiological compensatory mechanisms operating before the treatment. The physiological compensation could be biologically explained by a negative feedback mechanism mainly due to increased activation of the 5-HT1A autoreceptors present in the axonic terminals of 5-HT DRN neurons (Hajós et al., 1995). This mechanism produces an increase in DRN activity (Figures 3B, 6B up to 125 *s*). By contrast, the simulations run with the model to test the effect of the SSRIs treatment suggest that the negative feedback between the concentration of 5-HT and the DRN activity has to follow a different dynamical trajectory, producing a decrease in DRN activity to get a high DA level recovery. The reduction of the 5-HT degradation constant (τ_5−*HT*_) causes an increase in the 5-HT extracellular levels, which leads to an increased DA release by SNc. The effect is an excitatory modulation of DP and an inhibitory modulation of IP. This dynamic produces an increase in the firing rate of Thal and, in turn, of M1 with consequent reduction of the DRN activity.

Parkinson's disease tremor treatments based on dopamine medication (e.g., Levodopa administration) lead to a widespread and non-specific increase of DA concentration in the SNc (Connolly and Lang, 2014; Lee and Yankee, 2021). By contrast, the treatment with SSRIs proposed in this study would allow acting in specific ways on the area of interest. In this case, we increase the intrinsic concentration of 5-HT. By directing the drug delivery toward specific cellular markers (Huang et al., 2019), it is possible to inhibit only the SSRIs of our interest, allowing a more focused and not widespread increase in neurotransmitter concentration (Valentino and Commons, 2005; Wang et al., 2005; Rao and Nanda, 2009; Dankoski et al., 2016).

Despite these encouraging results, the current version of the model mainly represents a tool to test the effects of neurotransmission modulation rather than test new treatments based on 5-HT manipulation. Recent influential data demonstrate that 5-HT loss could precede PD motor symptoms and could be critical to study the PD pathophysiology and to develop novel therapeutic strategies (Wilson et al., 2019). However, evidence obtained from clinical research found several hurdles that, to date, have impaired the clinical development of serotonergic drugs (Huot et al., 2017; Muñoz et al., 2020). For example, the studies conducted in patients with PD and animal models mainly focussed only on 5-HT1A and 5-HT2A receptors, but the 5-HT system consists of many receptors (at least 14). It is not yet clear, what is the role of these other receptors. With 5-HT1A stimulation, it seems not easy to separate any benefit from interaction with L-DOPA anti-Parkinsonian action. In addition, the use of 5-HT1A receptor partial agonists also seems marred by a potentially detrimental effect on L-DOPA anti-Parkinsonian action, suggesting that such drugs may have a narrow therapeutic window (Bezard et al., 2013).

Hence, modulation of the 5-HT system is a promising way to address several manifestations of PD, but further investigation is required to clarify mechanisms of neurotransmitter interactions and to determine optimal compounds and doses for effective therapies producing the maximal benefit with minimal adverse events (Huot et al., 2017; Muñoz et al., 2020). New data from clinical research will support the design of an updated version of the model to make more consistent the simulations of the potential role of 5-HT for early diagnosis and therapy.

## 5. Conclusions

This article proposes a computational model to study how serotonin could affect dopamine release within the basal ganglia and how serotonin dysfunctions indirectly contribute to the emergence of tremor. The computer simulations run with the model suggest that serotonin changes monitoring could help in early PD diagnosis. They also demonstrate the effectiveness of possible new pharmacological treatments for tremor acting on serotonin to recover dopamine levels. However, evidence obtained from clinical research found several hurdles that, to date, have impaired the clinical development of serotonergic drugs (Huot et al., 2017; Muñoz et al., 2020). Therefore, more clinical evidence is needed to confirm or disprove the results obtained with the model.

The work focuses on the role of serotonin in Parkinsonian tremor, for this reason, studies how serotonin affects dopamine release through the meso-striatal circuits, including the SNc. Other studies on depression, mild cognitive impairment, and Alzheimer's disease, instead, have investigated the interaction between serotonin and dopamine release through circuits involving the meso-cortico-limbic network, including the ventral tegmental area (Martorana and Koch, 2014; Silvetti et al., 2019; Wang et al., 2019; Caligiore et al., 2020). Future studies on the relationship between these two different ways the serotonin affects dopamine could be critical to understanding the system-level neural mechanisms underlying the comorbidities between PD, depression, mild cognitive impairment, and Alzheimer's disease (Marsh, 2013; Caligiore et al., 2016, 2017b; Cummings et al., 2019).

## Data Availability Statement

The datasets presented in this study can be found in online repositories. The names of the repository/repositories and accession number(s) can be found at: https://github.com/ctnlab/serotonin_PD_tremor_model.

## Author Contributions

DC: conceptualization, formal analysis, investigation, methodology, resource, validation, software, visualization, writing - original draft, writing - review and editing, funding acquisition, project administration and supervision. FM: data curation, formal analysis, investigation, writing - review and editing, visualization, validation and software. SB: formal analysis, investigation, methodology, resource and software. AC: data curation, formal analysis, investigation, methodology, resource, writing - review and editing, visualization, validation and software. All authors contributed to the article and approved the submitted version.

## Conflict of Interest

The authors declare that the research was conducted in the absence of any commercial or financial relationships that could be construed as a potential conflict of interest.

## References

[B1] Alexander G. DeLong M. R. Strick P. L. (1986). Parallel organization of functionally segregated circuits linking basal ganglia and cortex. Ann. Rev. Neurosci. 9, 357–381. 10.1146/annurev.ne.09.030186.002041 3085570

[B2] Alvarez L. Macias R. Lopez G. Alvarez E. Pavon N. Rodriguez-Oroz M. C.. (2005). Bilateral subthalamotomy in parkinson's disease: initial and long-term response. Brain 128, 570–583. 10.1093/brain/awh397 15689366

[B3] An K. N. Hui F. C. Morrey B. F. Linscheid R. L. Chao E. Y. (1981). Muscles across the elbow joint: a biomechanical analysis. J. Biomech. 14, 659–669. 10.1016/0021-9290(81)90048-8 7334026

[B4] Bara-Jimenez W. Bibbiani F. Morris M. J. Dimitrova T. Sherzai A. Mouradian M. M.. (2005). Effects of serotonin 5-HT1A agonist in advanced Parkinson's disease. Mov. Disord. 20, 932–936. 10.1002/mds.20370 15791634

[B5] Bezard E. Tronci E. Pioli E. Y. Li Q. Porras G. Björklund A.. (2013). Study of the antidyskinetic effect of eltoprazine in animal models of levodopa-induced dyskinesia. Mov. Disord. 28, 1088–1096. 10.1002/mds.25366 23389842

[B6] Blandina P. Goldfarb J. Craddock-Royal B. Green J. P. (1989). Release of endogenous dopamine by stimulation of 5-hydroxytryptamine3 receptors in rat striatum. J. Pharmacol. Exper. Ther. 251(3):803–809. 2600815

[B7] Bolam J. P. Hanley J. J. Booth P. A. C. Bevan M. D. (2000). Synaptic organisation of the basal ganglia. J. Anat. 196, 527–542. 10.1046/j.1469-7580.2000.19640527.x 10923985 PMC1468095

[B8] Bonhomme N. De Deurwaèrdere P. Le Moal M. Spampinato U. (1995). Evidence for 5-HT4 receptor subtype involvement in the enhancement of striatal dopamine release induced by serotonin: a microdialysis study in the halothane-anesthetized rat. Neuropharmacology 34, 269–279. 10.1016/0028-3908(94)00145-I 7543190

[B9] Boraud T. Brown P. Goldberg J. A. Graybiel A. M. Magill P. J. (2005). Oscillations in the basal ganglia: the good, the bad, and the unexpected, in The Basal Ganglia VIII, eds Paul Bolam J. Ingham C. A. Magill P. J. (Boston, MA: Springer), 1–24.

[B10] Bosch-Bouju C. Hyland B. I. Parr-Brownlie L. C. (2013). Motor thalamus integration of cortical, cerebellar and basal ganglia information: implications for normal and parkinsonian conditions. Front. Comput. Neurosci. 7:163. 10.3389/fncom.2013.00163 24273509 PMC3822295

[B11] Cachope R. Cheer J. F. (2014). Local control of striatal dopamine release. Front. Behav. Neurosci. 8:188. 10.3389/fnbeh.2014.00188 24904339 PMC4033078

[B12] Caligiore D. Helmich R. C. Hallett M. Moustafa A. A. Timmermann L. Toni I.. (2016). Parkinson's disease as a system-level disorder. NPJ Parkinsons Dis. 2:16025. 10.1038/npjparkd.2016.25 28725705 PMC5516580

[B13] Caligiore D. Mannella F. Arbib M. A. Baldassarre G. (2017a). Dysfunctions of the basal ganglia-cerebellar-thalamo-cortical system produce motor tics in Tourette syndrome. PLoS Comput. Biol. 13:e1005395. 10.1371/journal.pcbi.1005395 28358814 PMC5373520

[B14] Caligiore D. Mannella F. Baldassarre G. (2019). Different dopaminergic dysfunctions underlying parkinsonian akinesia and tremor. Front. Neurosci. 13:550. 10.3389/fnins.2019.00550 31191237 PMC6549580

[B15] Caligiore D. Parisi D. Baldassarre G. (2014). Integrating reinforcement learning, equilibrium points, and minimum variance to understand the development of reaching: a computational model. Psychol. Rev. 121, 389–421. 10.1037/a0037016 25090425

[B16] Caligiore D. Pezzulo G. Baldassarre G. Bostan A. C. Strick P. L. Doya K.. (2017b). Consensus Paper: towards a systems-level view of cerebellar function: the interplay between cerebellum, basal ganglia, and cortex. Cerebellum 16, 203–229. 10.1007/s12311-016-0763-3 26873754 PMC5243918

[B17] Caligiore D. Pezzulo G. Miall R. C. Baldassarre G. (2013). The contribution of brain sub-cortical loops in the expression and acquisition of action understanding abilities. Neurosci. Biobehav. Rev. 37, 2504–2515. 10.1016/j.neubiorev.2013.07.016 23911926 PMC3878436

[B18] Caligiore D. Silvetti M. D'Amelio M. Puglisi-Allegra S. Baldassarre G. (2020). Computational modeling of catecholamines dysfunction in Alzheimer's disease at pre-plaque stage. J. Alzheimers Dis. 77, 275–290. 10.3233/JAD-200276 32741822 PMC7592658

[B19] Connolly B. S. Lang A. E. (2014). Pharmacological treatment of Parkinson disease: a review. J. Am. Med. Assoc. 311, 1670–1683. 10.1001/jama.2014.3654 24756517

[B20] Cummings J. Ritter A. Rothenberg K. (2019). Advances in management of neuropsychiatric syndromes in Neurodegenerative diseases. Curr. Psychiatry Rep. 21, 79. 10.1007/s11920-019-1058-4 31392434 PMC6685919

[B21] Dankoski E. C. Carroll S. Wightman R. M. (2016). Acute selective serotonin reuptake inhibitors regulate the dorsal raphe nucleus causing amplification of terminal serotonin release. J. Neurochem. 136, 1131–1141. 10.1111/jnc.13528 26749030 PMC4939133

[B22] De Deurwaerdère P. Bonhomme N. Lucas G. Le Moal M. Spampinato U. (2002). Serotonin enhances striatal dopamine outflow *in vivo* through dopamine uptake sites. J. Neurochem. 66, 210–215. 10.1046/j.1471-4159.1996.66010210.x 8522956

[B23] De La Fuente-Fernndez R. Lu J. Q. Sossi V. Jivan S. Schulzer M. Holden J. E.. (2001). Biochemical variations in the synaptic level of dopamine precede motor fluctuations in Parkinson's disease: PET evidence of increased dopamine turnover. Ann. Neurol. 49, 298–303. 10.1002/ana.65 11261503

[B24] De Simoni M. G. Dal Toso G. Fodritto F. Sokola A. Algeri S. (1987). Modulation of striatal dopamine metabolism by the activity of dorsal raphe serotonergic afferences. Brain Res. 411, 81–88. 10.1016/0006-8993(87)90683-4 2440514

[B25] Deuschl G. Raethjen J. Baron R. Lindemann M. Wilms H. Krack P. (2000). The pathophysiology of parkinsonian tremor: a review. J. Neurol. 247, V33–V48. 10.1007/PL00007781 11081802

[B26] Di Giovanni G. Di Matteo V. Esposito E. (2002). Serotonin/dopamine interaction-Focus on 5-HT2C receptor, a new target of psychotropic drugs. Indian J. Exper. Biol. 40, 1344–1352. 12974395

[B27] Di Matteo V. Pierucci M. Esposito E. Crescimanno G. Benigno A. Di Giovanni G. (2008). Serotonin modulation of the basal ganglia circuitry: therapeutic implication for Parkinson's disease and other motor disorders. Prog. Brain Res. 172, 423–463. 10.1016/S0079-6123(08)00921-7 18772045

[B28] Dogan O. Yuksel N. Ergun M. A. Yilmaz A. Ilhan M. N. Karslioglu H. E.. (2008). Serotonin transporter gene polymorphisms and sertraline response in major depression patients. Genet. Test. 12, 225–231. 10.1089/gte.2007.0089 18452396

[B29] Dovzhenok A. Rubchinsky L. L. (2012). On the origin of tremor in parkinson's disease. PLoS ONE 7:e41598. 10.1371/journal.pone.0041598 22848541 PMC3407214

[B30] Feldman R. S. Meyer J. S. Quenzer L. F. (1997). Principles of Neuropsychopharmacology. Sunderland, MA: Sinauer Associates Inc.

[B31] Fiore V. G. Mannella F. Mirolli M. Latagliata E. C. Valzania A. Cabib S.. (2015). Corticolimbic catecholamines in stress: a computational model of the appraisal of controllability. Brain Struct. Funct. 220, 1339–1353. 10.1007/s00429-014-0727-7 24578177 PMC4409646

[B32] Flash T. Hogan N. (1985). The coordination of arm movements: An experimentally confirmed mathematical model. J. Neurosci. 5, 1688–1703. 10.1523/JNEUROSCI.05-07-01688.1985 4020415 PMC6565116

[B33] Fortin F.-A. Marc-André Gardner U. Parizeau M. Gagné C. (2012). DEAP: evolutionary algorithms made easy. J. Mach. Learn. Res. 13, 2171–2175.

[B34] Gatev P. Darbin O. Wichmann T. (2006). Oscillations in the basal ganglia under normal conditions and in movement disorders. Mov. Disord. 21, 1566–1577. 10.1002/mds.21033 16830313

[B35] Gerfen C. R. Surmeier D. J. (2011). Modulation of striatal projection systems by dopamine. Ann. Rev. Neurosci. 34, 441–466. 10.1146/annurev-neuro-061010-113641 21469956 PMC3487690

[B36] Guiard B. P. El Mansari M. Merali Z. Blier P. (2008). Functional interactions between dopamine, serotonin and norepinephrine neurons: an *in-vivo* electrophysiological study in rats with monoaminergic lesions. Int. J. Neuropsychopharmacol. 11, 625–639. 10.1017/S1461145707008383 18205979

[B37] Guillery R. W. Bergman H. Feingold A. Nini A. Raz A. Slovin H.. (1998). Physiological aspects of information processing in the basal ganglia of normal and parkinsonian primates. Trends Neurosci. 2236, 32–38. 10.1016/S0166-2236(97)01151-X 9464684

[B38] Guillery R. W. Sherman S. M. (2002). The thalamus as a monitor of motor outputs. Philos. Trans. R. Soc. B Biol. Sci. 357, 1809–1821. 10.1098/rstb.2002.1171 PMC1693090 12626014

[B39] Gurney K. Prescott T. Redgrave P. (2001). A computational model of action selection in the basal ganglia. i. a new functional anatomy. Biol. Cybern. 84, 401–410. 10.1007/PL00007984 11417052

[B40] Haber S. N. (2003). The primate basal ganglia: parallel and integrative networks. J. Chem. Neuroanat. 26, 317–330. 10.1016/j.jchemneu.2003.10.003 14729134

[B41] Hajós M. Gartside S. E. Sharp T. (1995). Inhibition of median and dorsal raphe neurones following administration of the selective serotonin reuptake inhibitor paroxetine. Naunyn Schmiedebergs Arch. Pharmacol. 351, 624–629. 10.1007/BF00170162 7675121

[B42] Helmich R. C. (2018). The cerebral basis of Parkinsonian tremor: a network perspective. Mov. Dis. 33, 219–231. 10.1002/mds.27224 29119634

[B43] Helmich R. C. Hallett M. Deuschl G. Toni I. Bloem B. (2012). Cerebral causes and consequences of parkinsonian resting tremor: a tale of two circuits? Brain 135, 3206–3226. 10.1093/brain/aws023 22382359 PMC3501966

[B44] Hintzen A. Pelzer E. A. Tittgemeyer M. (2018). Thalamic interactions of cerebellum and basal ganglia. Brain Struct. Funct. 223, 569–587. 10.1007/s00429-017-1584-y 29224175

[B45] Huang K. W. Ochandarena N. E. Philson A. C. Hyun M. Birnbaum J. E. Cicconet M.. (2019). Molecular and anatomical organization of the dorsal raphe nucleus. Elife 8:e46464. 10.7554/eLife.46464 31411560 PMC6726424

[B46] Huot P. Sgambato-Faure V. Fox S. H. McCreary A. C. (2017). Serotonergic approaches in parkinson's disease: translational perspectives, an update. ACS Chem. Neurosci. 8, 973–986. 10.1021/acschemneuro.6b00440 28460160

[B47] Hutchison W. D. Lozano A. M. Tasker R. R. Lang A. E. Dostrovsky J. O. (1997). Identification and characterization of neurons with tremor-frequency activity in human globus pallidus. Exper. Brain Res. 113, 557–563. 10.1007/PL00005606 9108220

[B48] Jankovic J. (2018). Parkinson's disease tremors and serotonin. Brain 141, 624–626. 10.1093/brain/awx361 30063797

[B49] Jiménez-Sánchez L. Blesa J. Del Rey N. L. Monje M. H. Obeso J. A. Cavada C. (2019). Serotonergic innervation of the striatum in a nonhuman primate model of Parkinson's disease. Neuropharmacology 170, 107806. 10.1016/j.neuropharm.2019.107806 31589886

[B50] Jones S. R. Gainetdinov R. R. Jaber M. Giros B. Wightman R. M. Caron M. G. (1998). Profound neuronal plasticity in response to inactivation of the dopamine transporter. Proc. Natl. Acad. Sci. U.S.A. 95, 4029–4034. 10.1073/pnas.95.7.4029 9520487 PMC19957

[B51] Kalia L. V. Lang A. E. (2015). Parkinson's disease. Lancet 386, 896–912. 10.1016/S0140-6736(14)61393-3 25904081

[B52] Katayama M. Kawato M. (1993). Virtual trajectory and stiffness ellipse during multijoint arm movement predicted by neural inverse models. Biol. Cybern. 69, 353–362. 10.1007/BF01185407 8274536

[B53] Knobelman D. A. Hen R. Lucki I. (2001). Genetic regulation of extracellular serotonin by 5-hydroxytryptamin-1a and 5-hydroxytryptamin-1b autoreceptors in different brain regions of the mouse. J. Pharmacol. Exper. Ther. 298, 1083–1091. 11504805

[B54] Lee T. K. Yankee E. L. (2021). A review on Parkinson's disease treatment. Neuroimmunol. Neuroinflammation 8. 10.20517/2347-8659.2020.58

[B55] Lenz F. A. Kwan H. C. Martin R. L. Tasker R. R. Dostrovsky J. O. Lenz Y. E. (1994). Single unit analysis of the human ventral thalamic nuclear group. Brain 117, 531–543. 10.1093/brain/117.3.531 8032863

[B56] Levy R. Hutchison W. D. Lozano A. M. Dostrovsky J. O. (2000). High-frequency synchronization of neuronal activity in the subthalamic nucleus of parkinsonian patients with limb tremor. J. Neurosci. 20, 7766–7775. 10.1523/JNEUROSCI.20-20-07766.2000 11027240 PMC6772896

[B57] Lindgren H. S. Andersson D. R. Lagerkvist S. Nissbrandt H. Cenci M. A. (2010). L-DOPA-induced dopamine efflux in the striatum and the substantia nigra in a rat model of Parkinson's disease: temporal and quantitative relationship to the expression of dyskinesia. J. Neurochem. 112, 1465–1476. 10.1111/j.1471-4159.2009.06556.x 20050978

[B58] Lopes P. S. S. Campos A. C. P. Fonoff E. T. Britto L. R. G. Pagano R. L. (2019). Motor cortex and pain control: exploring the descending relay analgesic pathways and spinal nociceptive neurons in healthy conscious rats. Behav. Brain Funct. 15, 5. 10.1186/s12993-019-0156-0 30909927 PMC6432755

[B59] Mahon S. Vautrelle N. Pezard L. Slaght S. J. Deniau J. M. Chouvet G.. (2006). Distinct patterns of striatal medium spiny neuron activity during the natural sleep-wake cycle. J. Neurosci. 26, 12587–12595. 10.1523/JNEUROSCI.3987-06.2006 17135420 PMC6674897

[B60] Marreiros A. C. Cagnan H. Moran R. J. Friston K. J. Brown P. (2013). Basal ganglia-cortical interactions in Parkinsonian patients. Neuroimage 66, 301–310. 10.1016/j.neuroimage.2012.10.088 23153964 PMC3573233

[B61] Marsh L. (2013). Depression and parkinson's disease: Current knowledge topical collection on movement disorders. Curr. Neurol. Neurosci. Rep. 13, 409. 10.1007/s11910-013-0409-5 24190780 PMC4878671

[B62] Martorana A. Koch G. (2014). Is dopamine involved in Alzheimer's disease? Front. Aging Neurosci. 6:252. 10.3389/fnagi.2014.00252 25309431 PMC4174765

[B63] Middleton F. A. Strick P. L. (2000). Basal ganglia and cerebellar loops: motor and cognitive circuits. Brain Res. Rev. 31, 236–250. 10.1016/S0165-0173(99)00040-5 10719151

[B64] Mitchell K. T. Ostrem J. L. (2020). Surgical treatment of Parkinson disease. Neurol. Clin. 38, 293–307. 10.1016/j.ncl.2020.01.001 32279711

[B65] Monti J. M. (2011). Serotonin control of sleep-wake behavior. Sleep Med. Rev. 15, 269–281. 10.1016/j.smrv.2010.11.003 21459634

[B66] Mu noz A. Lopez-Lopez A. Labandeira C. M. Labandeira-Garcia J. L. (2020). Interactions between the serotonergic and other neurotransmitter systems in the basal ganglia: role in Parkinson's disease and adverse effects of L-DOPA. Front. Neuroanat. 14:26. 10.3389/fnana.2020.00026 32581728 PMC7289026

[B67] Murphy D. L. Lesch K. P. (2008). Targeting the murine serotonin transporter: insights into human neurobiology. Nat. Rev. Neurosci. 9, 85–96. 10.1038/nrn2284 18209729

[B68] Nambu A. Tokuno H. Takada M. (2002). Functional significance of the cortico-subthalamo-pallidal 'hyperdirect' pathway. Neurosci. Res. 43, 111–117. 10.1016/S0168-0102(02)00027-5 12067746

[B69] Nini A. Feingold A. Slovin H. Bergman H. (1995). Neurons in the globus pallidus do not show correlated activity in the normal monkey, but phase-locked oscillations appear in the MPTP model of parkinsonism. J. Neurophysiol. 74, 1800–1805. 10.1152/jn.1995.74.4.1800 8989416

[B70] Obeso J. A. Rodriguez-Oroz M. C. Goetz C. G. Marin C. Kordower J. H. Rodriguez M.. (2010). Missing pieces in the Parkinson's disease puzzle. Nat. Med. 16, 653–661. 10.1038/nm.2165 20495568

[B71] Ohara S. Taghva A. Kim J. H. Lenz F. A. (2007). Spontaneous low threshold spike bursting in awake humans is different in different lateral thalamic nuclei. Exper. Brain Res. 180, 281–288. 10.1007/s00221-007-0856-9 17256161

[B72] Özkurt T. E. Butz M. Homburger M. Elben S. Vesper J. Wojtecki L.. (2011). High frequency oscillations in the subthalamic nucleus: a neurophysiological marker of the motor state in Parkinson's disease. Exper. Neurol. 229, 324–331. 10.1016/j.expneurol.2011.02.015 21376039

[B73] Padovan-Neto F. E. Patterson S. F Voelkner N. M. Altwal F. Beverley J. A.. (2020). Selective regulation of 5-HT1B serotonin receptor expression in the striatum by dopamine depletion and repeated L-DOPA treatment: relationship to L-DOPA-induced dyskinesias. Mol. Neurobiol. 57, 736–751. 10.1007/s12035-019-01739-x 31468338 PMC7035192

[B74] Paraskevopoulou F. Herman M. A. Rosenmund C. (2019). Glutamatergic innervation onto striatal neurons potentiates GABAergic synaptic output. J. Neurosci. 39, 4448–4460. 10.1523/JNEUROSCI.2630-18.2019 30936241 PMC6554626

[B75] Pare D. Curro Dossi R. Steriade M. (1990). Neuronal basis of the parkinsonian resting tremor: a hypothesis and its implications for treatment. Neuroscience 35, 217–226. 10.1016/0306-4522(90)90077-H 2199839

[B76] Pasquini J. Ceravolo R. Qamhawi Z. Lee J.-Y. Deuschl G. Brooks D. J.. (2018). Progression of tremor in early stages of Parkinson's disease: a clinical and neuroimaging study. Brain 141, 811–821. 10.1093/brain/awx376 29365117

[B77] Planetta P. J. Schulze E. T. Geary E. K. Corcos D. M. Goldman J. G. Little D. M.. (2013). Thalamic projection fiber integrity in *de novo* Parkinson Disease. Am. J. Neuroradiol. 34, 74–79. 10.3174/ajnr.A3178 22766668 PMC3669594

[B78] Plenz D. Kital S. T. (1999). A basal ganglia pacemaker formed by the subthalamic nucleus and external globus pallidus. Nature 400, 677–682. 10.1038/23281 10458164

[B79] Politis M. Niccolini F. (2015). Serotonin in Parkinson's disease. Behav. Brain Res. 277, 136–145. 10.1016/j.bbr.2014.07.037 25086269

[B80] Qamhawi Z. Towey D. Shah B. Pagano G. Seibyl J. Marek K.. (2015). Clinical correlates of raphe serotonergic dysfunction in early Parkinson's disease. Brain 138, 2964–2973. 10.1093/brain/awv215 26209314

[B81] Rao R. Nanda S. (2009). Sonophoresis: recent advancements and future trends. J. Pharmacy Pharmacol. 61, 689–705. 10.1211/jpp.61.06.0001 19505359

[B82] Reed M. C. Nijhout H. F. Best J. (2013). Computational studies of the role of serotonin in the basal ganglia. Front. Integr. Neurosci. 7, 41. 10.3389/fnint.2013.00041 23745108 PMC3663133

[B83] Roussakis A.-A. Politis M. Towey D. Piccini P. (2016). Serotonin-to-dopamine transporter ratios in Parkinson disease. Neurology 86, 1152–1158. 10.1212/WNL.0000000000002494 26920358

[B84] Sargin D. Jeoung H.-S. Goodfellow N. M. Lambe E. K. (2019). Serotonin regulation of the prefrontal cortex: cognitive relevance and the impact of developmental perturbation. ACS Chem. Neurosci. 10, 3078–3093. 10.1021/acschemneuro.9b00073 31259523

[B85] Segovia G. Del Arco A. Mora F. (1997). Endogenous glutamate increases extracellular concentrations of dopamine, GABA, and taurine through NMDA and AMPA/Kainate receptors in striatum of the freely moving rat: a microdialysis study. J. Neurochem. 69, 1476–1483. 10.1046/j.1471-4159.1997.69041476.x 9326276

[B86] Serretti A. Calati R. Mandelli L. De Ronchi D. (2006). Serotonin transporter gene variants and behavior: a comprehensive review. Curr. Drug Targets 7, 1659–1669. 10.2174/138945006779025419 17168841

[B87] Sgambato-Faure V. Tremblay L. (2018). Dopamine and serotonin modulation of motor and non-motor functions of the non-human primate striato-pallidal circuits in normal and pathological states. J. Neural Transm. 125, 485–500. 10.1007/s00702-017-1693-z 28176009

[B88] Shimamoto S. A. Ryapolova-Webb E. S. Ostrem J. L. Galifianakis N. B. Miller K. J. Starr P. A. (2013). Subthalamic nucleus neurons are synchronized to primary motor cortex local field potentials in Parkinson's disease. J. Neurosci. 33, 7220–7233. 10.1523/JNEUROSCI.4676-12.2013 23616531 PMC3673303

[B89] Silvetti M. Baldassarre G. Caligiore D. (2019). A computational hypothesis on how serotonin regulates catecholamines in the pathogenesis of depressive apathy, in Multiscale Models of Brain Disorders (Cham: Springer), 127–134.

[B90] Smith Y. Bevan M. D. Shink E. Bolam J. P. (1998). Microcircuitry of the direct and indirect pathways of the basal ganglia. Neuroscience 86, 353–387. 9881853 10.1016/s0306-4522(98)00004-9

[B91] Stanford I. (2003). independent neuronal oscillators of the rat globus pallidus. J. Neurophysiol. 89, 1713–1717. 10.1152/jn.00864.2002 12626634

[B92] Stephenson-Jones M. Yu K. Ahrens S. Tucciarone J. M. Van Huijstee A. N. Mejia L. A.. (2016). A basal ganglia circuit for evaluating action outcomes. Nature 539, 289–293. 10.1038/nature19845 27652894 PMC5161609

[B93] Surmeier D. J. Mercer J. N. Chan C. S. (2005). Autonomous pacemakers in the basal ganglia: who needs excitatory synapses anyway? Curr. Opin. Neurobiol. 15, 312–318. 10.1016/j.conb.2005.05.007 15916893

[B94] Terman D. Rubin J. E. Yew A. C. Wilson C. J. (2002). Activity patterns in a model for the subthalamopallidal network of the basal ganglia. J. Neurosci. 22, 2963–2976. 10.1523/JNEUROSCI.22-07-02963.2002 11923461 PMC6758326

[B95] Timmermann L. Gross J. Dirks M. Volkmann J. Freund H. J. Schnitzler A. (2003). The cerebral oscillatory network of parkinsonian resting tremor. Brain 126, 199–212. 10.1093/brain/awg022 12477707

[B96] Valentino R. J. Commons K. G. (2005). Peptides that fine-tune the serotonin system. Neuropeptides 39, 1–8. 10.1016/j.npep.2004.09.005 15627494

[B97] Vertes R. P. (1991). A PHA-L analysis of ascending projections of the dorsal raphe nucleus in the rat. J. Compar. Neurol. 313, 643–668. 10.1002/cne.903130409 1783685

[B98] Wang H. L. Zhang S. Qi J. Wang H. Cachope R. Mejias-Aponte C. A.. (2019). Dorsal raphe dual serotonin-glutamate neurons drive reward by establishing excitatory synapses on vta mesoaccumbens dopamine neurons. Cell Rep. 26, 1128–1142. 10.1016/j.celrep.2019.01.014 30699344 PMC6489450

[B99] Wang Y. Thakur R. Fan Q. Michniak B. (2005). Transdermal iontophoresis: Combination strategies to improve transdermal iontophoretic drug delivery. Eur. J. Pharm. Biopharm. 60, 179–191. 10.1016/j.ejpb.2004.12.008 15939232

[B100] Wichmann T. DeLong M. R. (1999). Oscillations in the basal ganglia. Nature 400, 621–622. 10.1038/23148 10458157

[B101] Wilson H. Dervenoulas G. Pagano G. Koros C. Yousaf T. Picillo M.. (2019). Serotonergic pathology and disease burden in the premotor and motor phase of A53T α-synuclein parkinsonism: a cross-sectional study. Lancet Neurol. 18, 748–759. 10.1016/S1474-4422(19)30140-1 31229470

[B102] Wu T. Hallett M. (2013). The cerebellum in parkinson's disease. Brain 136, 696–709. 10.1093/brain/aws360 23404337 PMC7273201

[B103] Zach H. Dirkx M. F. Roth D. Pasman J. W. Bloem B. R. Helmich R. C. (2020). Dopamine-responsive and dopamine-resistant resting tremor in Parkinson disease. Neurology 95, e1461–e1470. 10.1212/WNL.0000000000010316 32651292

